# Prkci activates Jak2/Stat3 signaling to promote tumor angiogenesis: **Short Name**: Prkci in tumor angiogenesis

**DOI:** 10.1016/j.neo.2025.101219

**Published:** 2025-08-20

**Authors:** Peng Li, Guangshi Liu, Wenbin Zhang, Tao Li, Xinhui Yang

**Affiliations:** a Gastrointestinal Surgery department, People’s Hospital of Xinjiang Uygur Autonomous Region, Xinjiang, Urumqi 830000, China; b Department of Gastrointestinal Surgery, Xinjiang Medical University Affiliated Cancer Hospital, Gastrointestinal Surgery department, Xinjiang, Urumqi 830000, China

**Keywords:** Prkci, Colorectal cancer, Angiogenesis, Jak2/Stat3 pathway, Vegfa, Therapeutic target

## Abstract

**Background:**

Tumor angiogenesis is essential for colorectal cancer (CRC) progression, providing oxygen and nutrients to sustain tumor growth and metastasis. Protein kinase C iota (Prkci) is an atypical protein kinase known for its oncogenic roles in various cancers; however, its function in CRC angiogenesis remains largely unexplored. This study investigates the role of Prkci in regulating tumor angiogenesis through the Jak2/Stat3 signaling pathway.

**Methods:**

Prkci expression levels in CRC tissues and their correlation with micro-vessel density and patient prognosis were analyzed. Functional experiments, including endothelial cell proliferation, migration, and tube formation assays, were performed in vitro to assess the angiogenic effects of Prkci. In vivo, a CRC xenograft mouse model with Prkci knockout was used to evaluate tumor growth and angiogenesis. Mechanistic studies explored how Prkci activates Jak2 by phosphorylating it at the S633 site, leading to downstream Stat3 activation and Vegfa expression.

**Results:**

Prkci was upregulated in CRC tissues and correlated with increased micro-vessel density and poor patient prognosis. In vitro, Prkci overexpression enhanced endothelial cell proliferation, migration, and tube formation, while Prkci knockout inhibited these processes. Mechanistically, Prkci phosphorylated Jak2 at S633, leading to enhanced Stat3 activation and increased Vegfa expression, which promoted angiogenesis. In vivo, Prkci knockout in CRC cells significantly reduced tumor growth, angiogenesis, and prolonged survival in a mouse model.

**Conclusions:**

These findings identify Prkci as a key regulator of angiogenesis in CRC through Jak2/Stat3 signaling activation. Targeting Prkci could provide a novel therapeutic approach to inhibit tumor angiogenesis and limit CRC progression.

## Background

Tumor angiogenesis plays a pivotal role in cancer growth and progression. Tumor cells secrete various pro-angiogenic factors, including VEGF, PDGF, and FGF, to stimulate new blood vessel formation[1]. These new vessels are often irregular, permeable, and structurally abnormal, leading to inefficient blood flow and creating a hypoxic tumor microenvironment that further drives angiogenesis and tumor aggressiveness. Anti-angiogenic therapies aim to inhibit this process, thereby “starving” the tumor by cutting off its blood supply, which has been an effective therapeutic strategy in some kinds of cancer[[2], [3], [4]]. However, the efficacy of anti-angiogenic therapies is often limited by resistance mechanisms, such as alternative angiogenic pathways, vessel co-option, and hypoxia-Induced adaptation[[5], [6], [7], [8]]. Further elucidating the mechanisms of tumor angiogenesis may provide effective strategies to overcome treatment resistance.

Protein kinase C iota (Prkci) is an atypical protein kinase C family member known to play significant roles in cancer progression[[9], [10], [11]]. Its overexpression has been associated with enhanced cell proliferation, migration, and survival across various cancers, including osteosarcoma, pancreatic cancer, and cervical cancer. In osteosarcoma, Prkci promotes tumor cell growth by activating the Akt/mTOR pathway. Prkci also contributes to pancreatic cancer growth and metastasis through its interaction with RIPK2. In cervical cancer, Prkci’s overexpression is associated with poor prognosis and decreased sensitivity to radiotherapy via interacting with the Hedgehog/GLI1 [[12], [13], [14], [15]]. Prkci’s role in activating diverse oncogenic pathways makes it a crucial factor in tumor progression and treatment resistance, presenting opportunities for targeted cancer therapies. However, the biological function of Prkci in colorectal cancer is seldom studied, which is worthy to investigate.

Here, we identified Prkci as a pro-angiogenic factor in colorectal cancer, showing that it was upregulated in tumor tissues and associated with increased micro-vessel density, advanced clinical stage, and poorer survival outcomes. Mechanistically, Prkci promoted angiogenesis via activating the IL-6/Jak2/Stat3 signaling pathway; specifically, Prkci phosphorylated Jak2 at the S633 site, which was essential for IL-6/Jak2/Stat3 pathway activation. Additionally, in vivo experiments demonstrated that Prkci knockout in colorectal cancer cells reduced tumor growth, angiogenesis, and Jak2/Stat3 activation, significantly extending survival in a mouse model. Together, these findings suggested that targeting Prkci could offer a novel therapeutic strategy for colorectal cancer.

## Methods and material

### Cell lines and culture

Colorectal cancer cell lines HT29 and RKO were purchased from American Type Culture Collection (ATCC). HT29 were cultured in McCoy's 5a Medium, while RKO were cultured in Dulbecco's Modified Eagle Medium (DMEM). All mediums were supplemented with 10 % fetal bovine serum (FBS). Cells were cultured at 37°C in a humidified atmosphere with 5 % CO₂.

### Reagents

Antibodies for Prkci (66493, proteintech), β-actin (ab8226, abcam), Vegfa (ab52917, abcam), p-Jak2 (ab32101, abcam), Jak2 (ab108596, abcam), p-Stat3 (ab267373, abcam), Stat3 (ab68153, abcam), Na+/K+-ATPase (A11683, ABclonal), Gapdh (ab181602, abcam), pan Phospho-Serine/Threonine (AP1475, Abclonal), and CD31 (A19014, Abclonal) were bought from identified companies. IL-6 (HY-P7044) was bought from MedChemExpress company.

### Tube formation assay

Matrigel (CLS356234, Sigma-Aldrich) was thawed and evenly coated (50 µL) onto a 96-well plate, which was then incubated at 37°C for 30 minutes to solidify. HUVECs (ATCC) were prepared at a concentration of 2 - 4 × 10⁴ cells per 100 µL in different conditioned medias (CMs), then seeded onto the Matrigel-coated wells. After incubation at 37°C for 4–6 hours, tube formation was imaged and quantified by counting branching points and tube lengths using ImageJ software.

### Trans-well assay

Trans-well inserts (8 µm pore size) were placed in a 24-well plate with 600 µL CMs in the lower chamber. HUVECs (5 - 10 × 10⁴ cells in 100 µL serum-free media) were added to the upper chamber. After incubation at 37°C for 6–12 hours, non-migrated cells on the upper membrane surface were removed, and migrated cells were fixed with 4 % formaldehyde, stained with crystal violet, and rinsed. Migrated cells were imaged and counted using inverted optical microscope.

### CCK8 assay

HUVECs were seeded in a 96-well plate at 2 - 5 × 10³ cells per well in 100 µL CMs. After incubation at 37°C for 24, 48, or 72 hours, CCK-8 solution was added to each well and incubated for 1–2 more hours. Absorbance was then measured at 450 nm to assess cell proliferation using microplate reader.

### Real-time PCR

Total RNAs were extracted from cells using TRIzol™ Reagent (Thermo Fisher) , and cDNA was synthesized. Real-time PCR was performed using specific primers and SYBR Green master mix (RK21203, Abclonal), with samples run in triplicate. Relative gene expression was calculated with the ΔΔCt method, normalized to a housekeeping gene. The primers (PrimerBank database) [16] were listed as followed: Vegfa F: 5- AGGGCAGAATCATCACGAAGT-3, Vegfa R: 5- AGGGTCTCGATTGGATGGCA-3, beta actin: F: 5-CATGTACGTTGCTATCCAGGC-3, beta actin R: 5- CTCCTTAATGTCACGCACGAT-3, Ang2 F: 5-AACTTTCGGAAGAGCATGGAC-3, Ang2 R: 5-CGAGTCATCGTATTCGAGCGG-3, bFGF F: 5- AGAAGAGCGACCCTCACATCA-3, bFGF R: 5- CGGTTAGCACACACTCCTTTG-3, Il8 F: 5- TTTTGCCAAGGAGTGCTAAAGA-3, Il8 R: 5-AACCCTCTGCACCCAGTTTTC-3, Mmp9 F: 5- TGTACCGCTATGGTTACACTCG-3, Mmp9 R: 5- GGCAGGGACAGTTGCTTCT-3, Vegfb F: 5-GAGATGTCCCTGGAAGAACACA-3, Vegfb R: 5- GAGTGGGATGGGTGATGTCAG-3.

### ELISA assay

Human Vegfa ELISA Kit (RK00023) was purchased from ABclonal company. CMs were collected from different cancer cells. The concentration of Vegfa was measured using Human Vegfa ELISA Kit.

### Western blotting and immunoprecipitation

Cells were lysed using NP-40 lysis buffer, and proteins were extracted, quantified, and separated by SDS-PAGE. Proteins were then transferred to a PVDF membrane, blocked, and incubated with primary and secondary antibodies. Protein bands were visualized using ECL substrate and imaged to assess protein expression. For immunoprecipitation, cells were lysed, and lysates were pre-cleared with protein A/G beads. After incubating with the primary antibody, fresh beads were added to capture antibody-protein complexes. Beads were washed, and bound proteins were eluted with SDS buffer, followed by analysis via Western blot.

### Immunohistochemistry (IHC)

Tissue sections were deparaffinized, rehydrated, and underwent antigen retrieval. Blocking was followed by incubation with the primary antibody overnight, then a secondary antibody and DAB for visualization. Sections were counterstained with hematoxylin, dehydrated, and mounted to observe staining patterns. For IHC scoring, staining intensity was rated from 0 (none) to 3 (strong), and the percentage of positive cells was scored from 0 (0 %) to 4 (76–100 %). The overall score was calculated by multiplying the intensity and percentage scores, resulting in a range of 0 to 12. Scores were then categorized as low (0–7), high expression [[8], [9], [10], [11], [12]] to quantify protein levels in tissue samples.

### Subcutaneous tumor xenograft assay

colorectal cancer cells were cultured to 80-90 % confluency, harvested, washed with PBS, and resuspended in serum-free medium. Immunodeficient mice (6-8 weeks old, BALB/c-Nude, Strain NO D000521, GemPharmatech Co., Ltd) were anesthetized, and 1 × 10⁶ cells in 100 µL were injected subcutaneously into the right flank. Tumor growth was monitored every 2 days by measuring length (L) and width (W) with calipers, and volume was calculated using the formula: Volume=*L*×*W*^2^/2. At the experimental endpoint, mice were humanely euthanized, tumors were excised and weighed, and samples were prepared for further analyses such as immunohistochemistry (IHC) or Western blot to evaluate protein expression and molecular markers. All animal procedures were conducted according to institutional guidelines and approved by the animal ethics committee of People Hospital of Xinjiang Uygur Autonomous Region.

### Statistical analysis

Data was collected and organized using GraphPad Prism 9. Data distribution was assessed. Parametric tests (t-tests) and non-parametric tests (Mann-Whitney U test) were applied. A significance level of p<0.05. p<0.05 and 95 % confidence intervals were used to determine statistical significance. Results were reported as mean ± SD. These statistical analyses provided a rigorous evaluation, supporting interpretation and discussion of the experimental findings.

## Result

### Prkci might act as a pro-angiogenesis factor in colorectal cancer

**Fig. 1:**
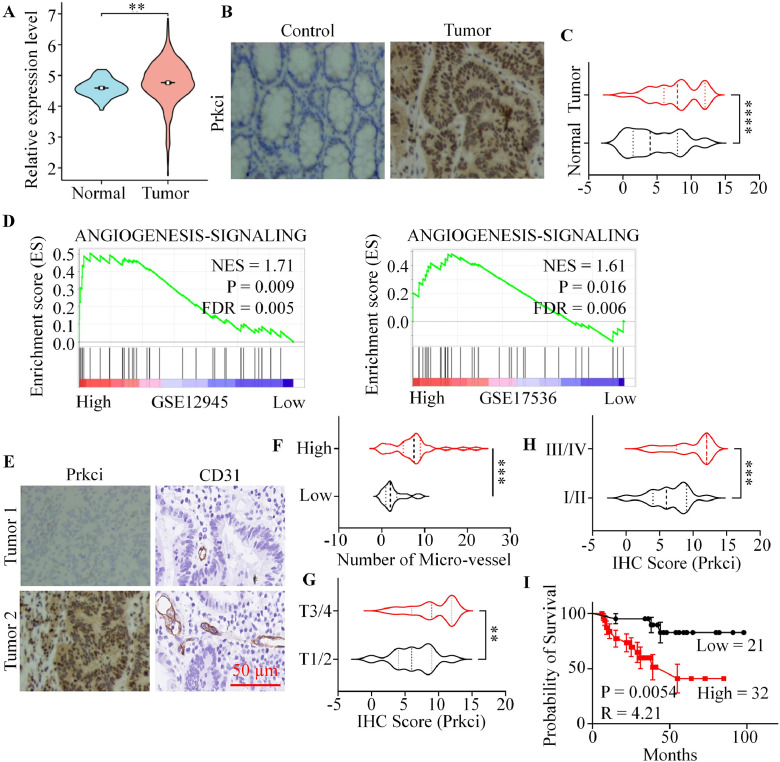
Prkci expression was associated with angiogenesis and poor clinical outcomes in colorectal cancer. (A) Compared with the expression levels of Prkci in normal and colorectal tumor tissues using TCGA database. (B) Representative immunohistochemical staining for Prkci in normal and colorectal tumor tissues. (C) Violin plot comparing Prkci expression between normal and colorectal tumor tissues. (D) Gene Set Enrichment Analysis (GSEA) for angiogenesis signaling pathways in CRC datasets (GSE12945 and GSE17536). (E) Immunohistochemistry for Prkci and CD31 in two representative CRC tumor samples. (F) Violin plot comparing micro-vessel density in tumors with high and low Prkci expression. (G) Violin plot of Prkci immunohistochemical score in different tumor stages (T1/2 vs. T3/4). (H) Prkci immunohistochemical score across clinical stages (I/II vs. III/IV). (I) Kaplan-Meier survival curve showing that patients with high Prkci expression have significantly poorer survival outcomes. Statistical analysis was conducted using Student's t-test.

To examine the impact of Prkci on tumor angiogenesis, we firstly analyzed the expression level of Prkci in normal tissues and colorectal cancer tissues using TCGA database. Statical analysis showed that Prkci was significantly upregulated in colorectal cancer compared with normal tissues (Fig. 1A). Also, Prkci was overexpressed in other kinds of tumor tissues compared with normal tissues (Figure S1A). Immunohistochemical (IHC) staining was also performed on 53 pairs of normal and colorectal tumor tissue samples. The results indicated a significantly elevated expression of Prkci in tumor tissues compared to normal tissues (Fig. 1B, 1C). Of note, Gene set enrichment analysis (GSEA) using multiple datasets demonstrated that high Prkci expression was significantly associated with the upregulation of angiogenesis-related signaling pathways (Fig. 1D and Figure S1B). Subsequently, we used CD31 antibody to mark the micro-vessel in colorectal cancer tissues. Tumors were grouped by the expression level of Prkci (IHC score no >6 was thought low). Results showed that tumors with high Prkci expression had higher CD31-positive micro-vessel density (Fig. 1E, 1F). Meanwhile, patients with higher T and clinical stage had higher Prkci expression level (Fig. 1G, 1H). Survival analysis was conducted to assess the clinical relevance of Prkci expression in CRC. Kaplan-Meier analysis revealed that patients with high Prkci expression had significantly reduced overall survival (OS) compared to those with low expression (Fig. 1I). Similar results were found in other kinds of tumors (Figure S1C). These findings suggested that Prkci activation was strongly linked to increased angiogenesis within the tumor microenvironment.

### Prkci positively regulated angiogenesis in vitro

**Fig. 2:**
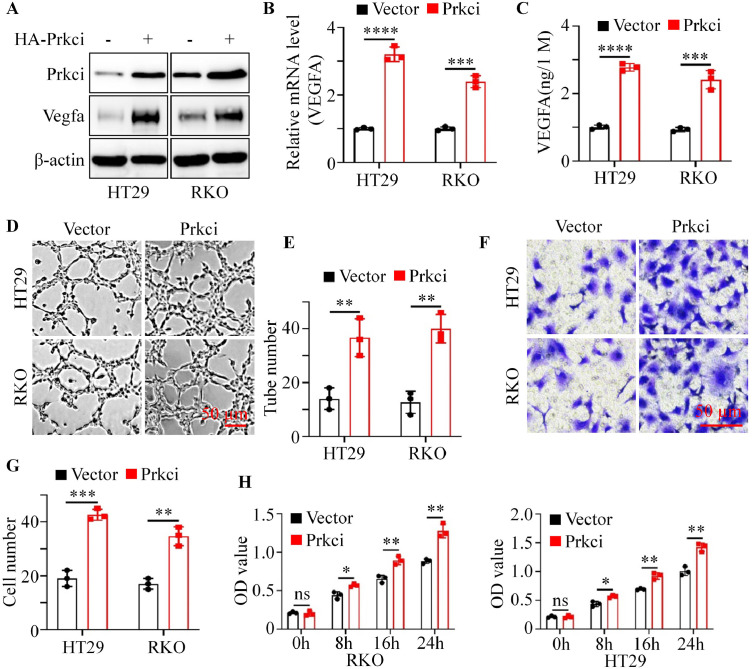
Overexpression of Prkci promoted Vegfa expression and angiogenesis-related functions in colorectal cancer cells. (A) Western blot analysis showed the expression of Prkci and Vegfa in vector and Prkci cells. (B) Relative mRNA levels of Vegfa in vector and Prkci cells were measured by qPCR. (C) ELISA results indicated elevated secretion of Vegfa protein in the culture media of Prkci cells compared to vector cells. (D) Representative images from tube formation assays. (E) Quantification of tube number from Fig. 2D. (F) Representative images from trans-well assays. (G) Quantification of migrated cells from Fig. 2F. (H) CCK8 assays showed the proliferation ability of HUVECs at various time points. Each IB assay was performed in triplicate, yielding consistent results. Statistical analysis was conducted using Student's t-test.

To assess the impact of Prkci angiogenesis, colorectal cancer cell lines HT29 and RKO were engineered to overexpress Prkci. Vegfa was an essential factor for tumor angiogenesis. Hence, we detected the Vegfa expression level in vector and Prkci-overexpressing cancer cells. Western blot analysis confirmed that Prkci overexpression significantly increased Vegfa protein levels in both HT29 and RKO cells compared to vector control cells (Fig. 2A). Quantitative PCR analysis demonstrated a corresponding increase in Vegfa mRNA levels in Prkci-overexpressing cells (Fig. 2B), and ELISA results indicated a significant elevation of Vegfa secretion in Prkci-overexpressing cells (Fig. 2C). Additionally, a tube formation assay with HUVECs demonstrated that conditioned medias (CMs) from Prkci-overexpressing CRC cells significantly promoted tube formation, with a marked increase in the number of branching points, indicating enhanced angiogenic activity (Fig. 2D, 2E). Next, we used CMs from vector and Prkci-overexpressing CRC cells to carry out trans-well assays with HUVECs. Trans-well assays revealed that CMs from Prkci-overexpressing CRC cells significantly promoted HUVECs migration ability (Fig. 2F, 2G). CCK8 assays demonstrated that CMs from Prkci-overexpressing CRC cells obviously promoted HUVECs proliferation ability compared to CMs from vector CRC cells (Fig. 2H).

**Fig. 3:**
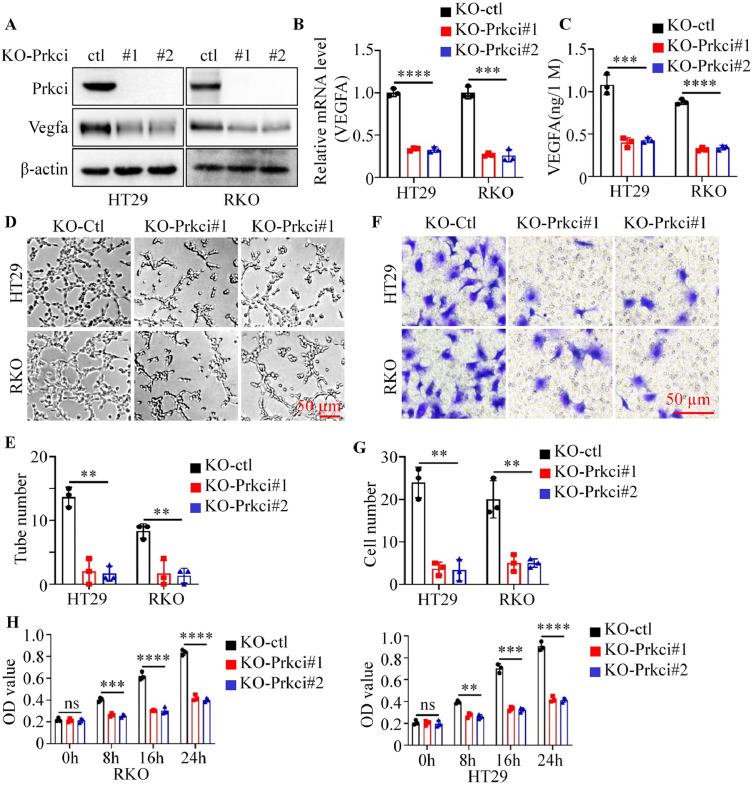
Knockout of Prkci reduced Vegfa expression and angiogenesis-related functions in colorectal cancer cells. (A) Western blot analysis showed the expression of Prkci and Vegfa in KO-ctl and KO-Prkci cells. (B) Relative mRNA levels of Vegfa in KO-ctl and KO-Prkci cells were measured by qPCR. (C) ELISA results indicated decreased secretion of Vegfa protein in the culture media of KO-Prkci cells compared to KO-ctl cells. (D) Representative images from tube formation assays. (E) Quantification of tube number from Fig. 3D. (F) Representative images from trans-well assays. (G) Quantification of migrated cells from Fig. 3F. (H) CCK8 assays showed the proliferation ability of HUVECs at various time points. Each IB assay was performed in triplicate, yielding consistent results. Statistical analysis was conducted using Student's t-test.

To further investigate Prkci’s role, knockout experiments for Prkci were performed in HT29 and RKO cells using crisp-cas9 system. Prkci knockout led to a substantial decrease in Vegfa expression, as confirmed by western blot, RT-PCR and ELISA (Fig. 3A-C). HUVECs exposed to CMs from KO-Prkci cells exhibited significantly fewer tube formations compared to those exposed to CM from control cells (KO-ctl) (Fig. 3D-E). Trans-well migration assay showed that CMs from KO-Prkci cells led to a significant reduction in the number of migrated HUVECs (Fig. 3F-G). CMs from KO-Prkci cells induced a marked reduction in proliferation of HUVECs compared to control cells (Fig. 3H). These results suggested that Prkci positively stimulated angiogenesis in the tumor microenvironment.

### Prkci regulated IL-6/Jak2/Stat3 signaling pathway in CRC cells

**Fig. 4:**
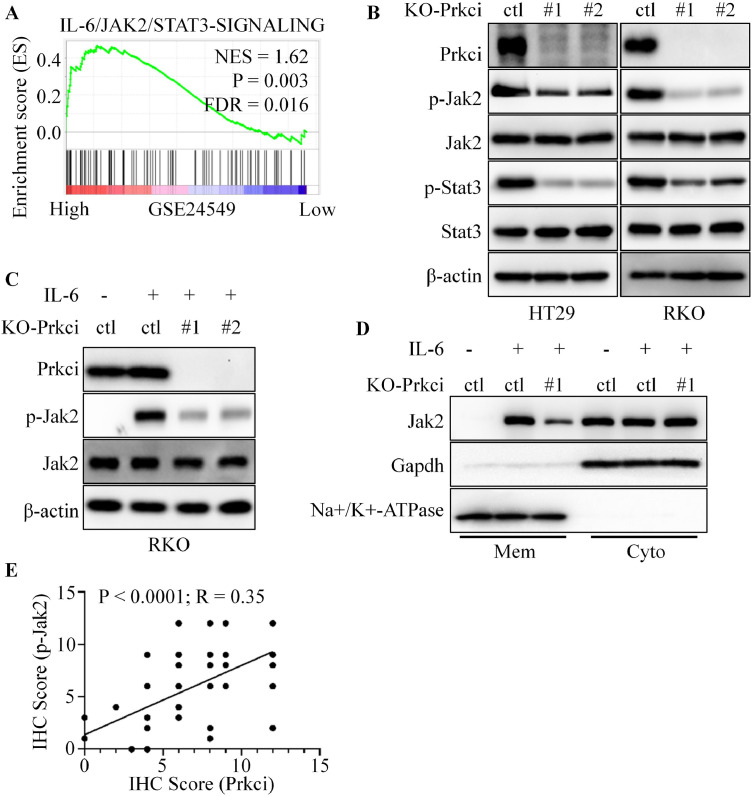
Prkci regulated the IL-6/Jak2/Stat3 signaling pathway in colorectal cancer cells. (A) Gene Set Enrichment Analysis (GSEA) indicated that high Prkci expression was positively associated with the activation of the IL-6/Jak2/Stat3 signaling pathway. (B) Western blot analysis showed that KO-Prkci cells reduced the levels of p-Jak2 and p-Stat3 compared to KO-ctl cells. (C) Western blot of RKO cells indicated that IL-6-induced phosphorylation of Jak2 was reduced in KO-Prkci cells compared to KO-ctl cells. (D) Membrane and cytosolic fractionation analysis demonstrated that IL-6 treatment promoted membrane localization of Jak2, while Prkci knockout reversed this effect. (E) Immunohistochemistry (IHC) analysis revealed a positive correlation between Prkci and p-Jak2 expression in clinical CRC samples. Each IB assay was performed in triplicate, yielding consistent results. Statistical analysis was conducted using Student's t-test.

To investigate the mechanism by which Prkci influences angiogenesis, gene set enrichment analysis (GSEA) was performed and demonstrated that high Prkci expression was significantly associated with activation of the IL-6/Jak2/Stat3 signaling pathway (Fig. 4A and Figure S2A). This suggested that Prkci might regulate key components of this pathway, which was known to play a role in tumor angiogenesis. Western blot analysis showed a substantial decrease in phosphorylated Jak2 (p-Jak2) and phosphorylated Stat3 (p-Stat3) levels in KO-Prkci cells (Fig. 4B). Also, Prkci overexpression significantly increased the phosphorylation of Jak2 (Figure S2B). The total protein levels of Jak2 and Stat3 were unaffected, indicating that Prkci specifically impacted the activation (phosphorylation) of these signaling molecules. Further analysis was performed to assess the effect of IL-6 stimulation on Jak2 phosphorylation in Prkci knockout RKO cells. Western blot results showed that IL-6-induced phosphorylation of Jak2 was reduced in KO-Prkci cells compared to control cells (Fig. 4C). Consistently, Prkci overexpression could further enhance IL-6-induced Jak2 phosphorylation (Figure S2C). We also examined the subcellular localization of Jak2 in KO-ctl and KO-Prkci cells under IL-6 stimulation. Membrane and cytosolic fractionation indicated that the IL-6 application significantly increased the membrane localization of Jak2, while Prkci knockout reversed the result and Prkci overexpression enhanced the effect (Fig. 4D and figure S2D). To further investigate the relationship between Prkci expression and Jak2 activation in clinical samples, immunohistochemistry (IHC) for Prkci and p-Jak2 were analyzed. A positive correlation was observed between Prkci and p-Jak2 expression (Fig. 4E and figure S2E). Moreover, PRKCI knockdown suppressed the expression of several known pro-angiogenic genes downstream of STAT3, including Ang-2, bFGF, Il-8, Mmp9, and Vegfb (Figure S2F). In summary, these results suggested that Prkci enhanced the activation of the IL-6/Jak2/Stat3 signaling pathway, likely through promoting Jak2 phosphorylation and membrane localization.

### Prkci phosphorylated Jak2 through the S633 site

**Fig. 5:**
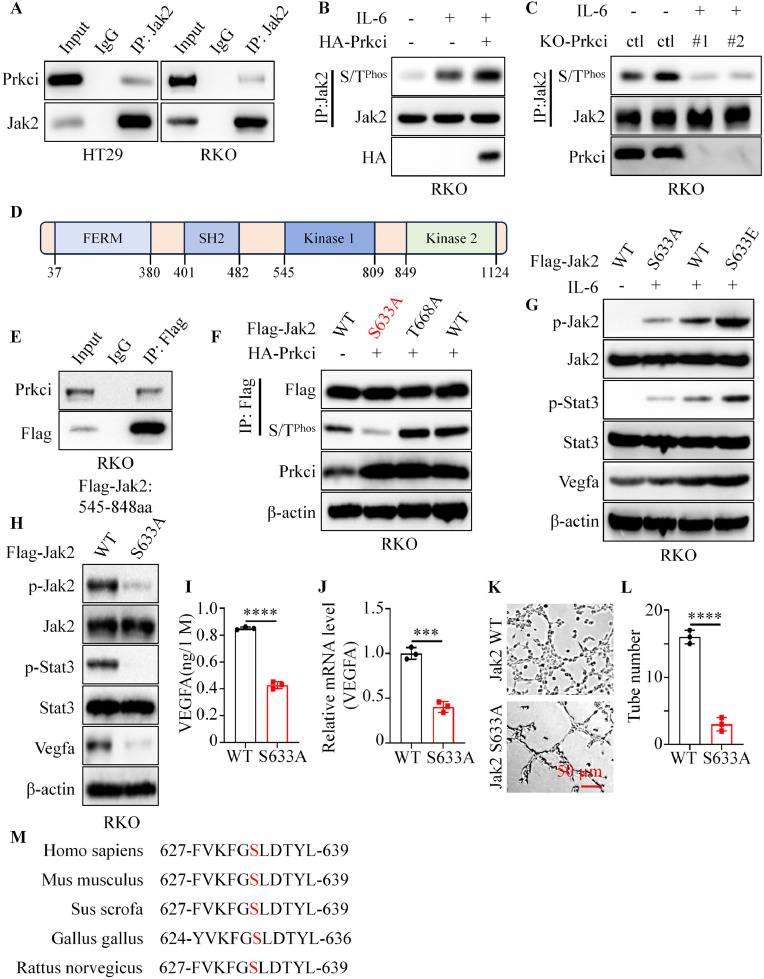
Prkci phosphorylated Jak2 at S633, enhancing Jak2/Stat3 signaling. (A) Co-immunoprecipitation (Co-IP) analysis showed that Prkci interacted with Jak2. (B) Western blot analysis indicated that Prkci overexpression enhanced IL-6-induced S/T^Phos^ of Jak2. (C) In KO-Prkci cells, IL-6-induced Jak2 S/T^Phos^ was significantly reduced compared to KO-ctl cells. (D) Schematic representation of Jak2 domains. (E) Co-IP confirmed the binding of Prkci and the 545-848 amino acid region of Jak2. (F) Western blot showed that Prkci phosphorylated Jak2 at S633, as S633A (mutation to alanine) abolished phosphorylation, while T668A did not affect it. (G) Western blot showed that the S633A mutation led to reduced phosphorylation of Jak2 and Stat3, along with decreased Vegfa expression, in response to IL-6 stimulation, while S633E mutation had the opposite results. (H) Jak2 S633A mutation significantly decreased p-Jak2, p-Stat3, and Vegfa levels. (I, J) ELISA and qPCR analyses demonstrated lower Vegfa protein and mRNA levels in cells with Jak2 S633A mutation. (K) Representative tube formation assay images. (L) Quantification of tube formation assays form Fig. 5K. (M) Sequence alignment showed the conservation of the S633 site across multiple species. Each IB assay was performed in triplicate, yielding consistent results. Statistical analysis was conducted using Student's t-test.

To investigate the mechanism by which Prkci modulated Jak2 activation, we firstly examined the interaction between Prkci and Jak2. Co-immunoprecipitation assays showed that Prkci bound with Jak2, but not Stat3, in both HT29 and RKO cells (Fig. 5A and figure S3A). Considering that Prkci acted as a calcium- and diacylglycerol-independent serine/ threonine-protein kinase, we hypothesized that Prkci might directly phosphorylate Jak2. Prkci overexpression further increased IL-6-induced Jak2 phosphorylation at serine/threonine residues (S/T^Phos^) (Fig. 5B and figure S3B). Also, knockout of Prkci led to a marked reduction in IL-6-induced Jak2 serine/threonine phosphorylation (Fig. 5C and figure S3C).

To determine the specific region of Jak2 required for Prkci interaction, Jak2 various segments were generated according to the domains of Jak2. CO-IP assays showed that Jak2 545-848 aa fragment was essential for the binding of Jak2 and Prkci (Fig. 5D, 5E and figure S3D). After browsing the PhosphoSitePlus database, there were two potential phosphorylation sites (S633 and T668). Then, we substituted these animo with alanine, which simulated phosphorylation deletion mutation. Interestingly, Prkci overexpression significantly increased the phosphorylation level of Jak2 WT and T668A, but not Jak2 S633A, which suggested that Prkci phosphorylated Jak2 at S633 (Fig. 5F). Next, we generated Jak2 WT, Jak2 S633A, or Jak2 S633E RKO cells. We applied IL-6 to these cells. IL-6 application could significantly increase the level of p-Jak2, p-Stat3, and Vegfa in Jak2 WT, while Jak2 S633A mutant reversed these changes and Jak2 S633E mutant reinforced these changes (Fig. 5G). Further analysis was conducted to assess the impact of the S633A mutation on downstream signaling. Western blot results indicated that cells expressing Jak2 S633A exhibited reduced phosphorylation of both Jak2 and Stat3, as well as decreased Vegfa expression, compared to cells expressing Jak2 WT (Fig. 5H). ELISA and RT-PCR confirmed a significant reduction in Vegfa protein secretion and mRNA levels in cells with the S633A mutation (Fig. 5I, 5J). HUVECs exposed to media from Jak2 S633A cells displayed significantly reduced tube formation compared to those exposed to media from Jak2 WT cells (Fig. 5K, 5L). Sequence alignment across species revealed that the serine residue at position 633 in Jak2 is highly conserved, suggesting its functional importance (Fig. 5M). In summary, these results indicated that Prkci enhanced Jak2 activation through phosphorylating Jak2 at S633 site.

### The S633 phosphorylation of Jak2 was essential for Prkci-mediated activation of the Jak2/Stat3 pathway and angiogenesis

To further investigate the functional relevance of the S633 phosphorylation of Jak2, we analyzed its role in Prkci-mediated Jak2/Stat3 activation, Vegfa expression, cell migration, and angiogenesis. Firstly, western blot analysis demonstrated that knockdown of Jak2 or inhibition of its activation with Jak2-IN-6 led to a significant reduction in Prkci-mediated increases in Stat3 phosphorylation and Vegfa expression (Figure S4A-B). Next, we introduced the activating mutation Jak2 V617F into Prkci knockdown cells. Results showed that Jak2 V617F partially restored Stat3 phosphorylation and Vegfa expression in the absence of Prkci (Figure S4C). Trans-well assays revealed that cells expressing Jak2 WT with Prkci knockdown exhibited significantly reduced migration, while the Jak2 V617F mutation restored migration capacity to levels comparable to control cells (Figure S4D-E). These results showed that Jak2 was an essential factor for Prkci-mediated activation of the Il-6/Jak2/Stat3 signaling.

**Fig. 6:**
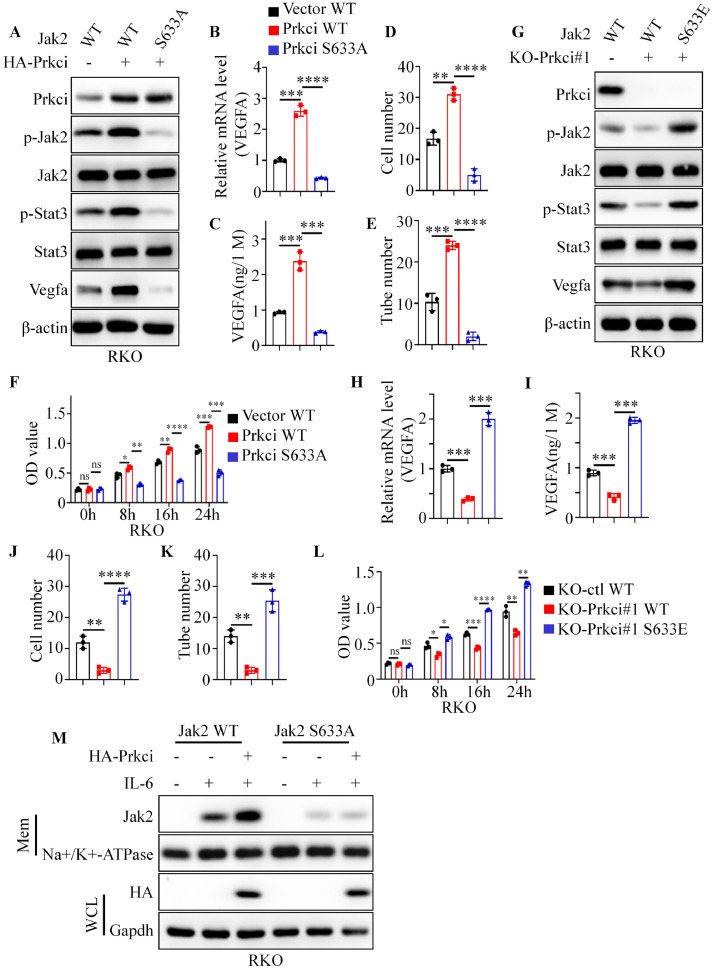
Prkci-induced Jak2 S633 phosphorylation was an essential factor for Prkci-mediated tumor angiogenesis. (A, G) Western blot analysis showed the expression level of multiple proteins in different cells. (B, H) Quantitative PCR detected the mRNA level of Vegfa in different cells. (C, I) ELISA detected the level of Vegfa protein in different CMs. (D, J) Statistical analysis for trans-well assays of HUVECs cultured with different CMs. (E, K) Statistical analysis for tube formation assays of HUVECs cultured with different CMs. (F, L) CCK-8 assays showed the proliferation of HUVECs in the presence of different CMs. (M) Membrane and cytosolic fractionation analysis detected the membrane localization of Jak2 in different cells. Each IB assay was performed in triplicate, yielding consistent results. Statistical analysis was conducted using Student's t-test.

Next, western blot, qPCR and ELISA results revealed that Prkci overexpression significantly increased phosphorylation of Jak2 and Stat3, as well as Il-6/Jak2/Stat3-targeting genes expression, in cells expressing Jak2 WT. In contrast, the Jak2 S633A mutant displayed markedly reduced phosphorylation of Jak2 and Stat3, as well as lower Il-6/Jak2/Stat3-targeting genes levels (Fig. 6A-C and figure S4F). Trans-well migration assays showed that Prkci overexpression in cells with Jak2 WT significantly increased cell migration, while cells with the Jak2 S633A mutation reversed the Prkci overexpression-mediate migratory capacity (Fig. 6D and figure S4G). Similarly, HUVEC tube formation assays revealed a significant increase in tube formation with CMs from Jak2 WT cells overexpressing Prkci, while Jak2 S633A reversed the tube formation ability (Fig. 6E and figure S4H). Next, HUVECs cultured with CMs from Prkic + Jak2 WT cells enhanced its proliferation ability compared to HUVECs cultured with CMs from vector+ Jak2 WT cells, while Jak2 S633A mutant reversed the effect of Prkci overexpression (Fig. 6F).

Subsequently, we generated RKO cells expressing KO-ctl + Jak2 WT, KO-Prkci + Jak2 WT, KO-Prkci + Jak2 S633E. ELISA, qPCR and Western blot analysis indicated that the Jak2 S633E mutant restored Jak2 and Stat3 phosphorylation, as well as Vegfa expression, in Prkci knockdown cells (Fig. 6G-I). Additionally, migration, tube formation and CCK8 assays demonstrated that cells with the S633E mutation restored migration and angiogenic capabilities in Prkci knockdown cells (Fig. 6J-L and figure S4I, S4J). Finally, the subcellular localization of Jak2 WT and S633A was examined in the presence of Prkci or IL-6. Membrane fractionation assays indicated that Prkci and IL-6 promoted membrane localization of Jak2 WT but not Jak2 S633A, suggesting that S633 phosphorylation was essential for Jak2’s proper localization and function (Fig. 6M). In summary, these results indicated that the S633 phosphorylation on Jak2 was essential for Prkci-mediated activation of the Jak2/Stat3 pathway, Vegfa expression, cell migration, angiogenesis, and cell proliferation.

### Targeting Prkci reduced tumor growth, angiogenesis, and increased survival in a mouse xenograft model

**Fig. 7:**
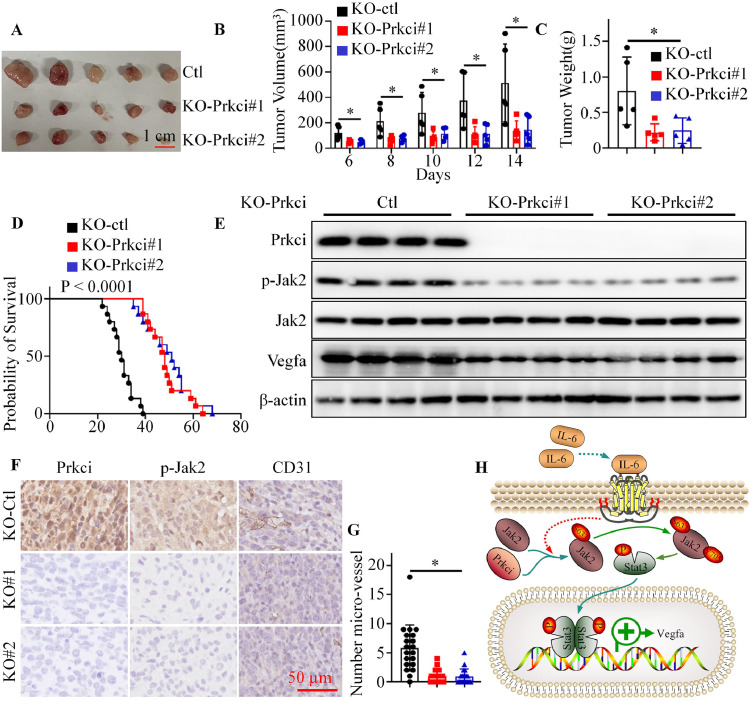
Prkci knockout reduced tumor growth, angiogenesis, and improved survival in a colorectal cancer xenograft model. (A) Representative images of tumors from mice injected with KO-ctl and KO-Prkci colorectal cancer cells. (B) Tumor volume was significantly reduced in the KO-Prkci groups compared to KO-ctl. (C) Tumor weight was significantly lower in the KO-Prkci groups. (D) Kaplan-Meier survival analysis showed a significantly prolonged survival in mice bearing KO-Prkci tumors compared to controls. (E) Western blot analysis of tumor tissues showed reduced levels of phosphorylated Jak2 and Vegfa in KO-Prkci tumors. (F) Representative images of immunohistochemical staining for Prkci, p-Jak2, and CD31. (G) Quantification of CD31-positive micro-vessels from Fig. 7F. (H) The working model: Prkci phosphorylated Jak2 at S633 to promote IL-6/Jak2/Stat3 signaling activation and tumor angiogenesis. Each IB assay was performed in triplicate, yielding consistent results. Statistical analysis was conducted using Student's t-test.

To investigate the effect of Prkci on tumor growth and angiogenesis in vivo, we utilized a mouse xenograft model with Prkci knockout colorectal cancer cells. Tumors derived from Prkci knockout cells exhibited significantly smaller sizes and weights compared to control tumors (Fig. 7A-C). Kaplan-Meier survival analysis demonstrated that mice bearing Prkci knockout tumors had a significantly prolonged survival compared to control mice (Fig. 7D). Western blot analysis of tumor tissues showed a marked decrease in phosphorylated Jak2 and Stat3, as well as reduced Vegfa expression, in Prkci knockout tumors compared to controls (Fig. 7E). Immunohistochemical staining for Prkci, p-Jak2, and the endothelial marker CD31 revealed a substantial decrease in micro-vessel density and Jak2 phosphorylation in Prkci knockout tumors compared to controls (Fig. 7F, 7G). These findings suggested that targeting Prkci might offer therapeutic potential in colorectal cancer by impairing tumor growth and angiogenesis.

## Discussion

The findings from this study established Prkci as a pro-angiogenic factor in CRC by activating the Jak2/Stat3 signaling pathway through phosphorylation of Jak2 at S633. The role of angiogenesis in tumor biology was well documented, with tumor cells often relying on an oxygen-deprived microenvironment to induce the secretion of pro-angiogenic factors like Vegfa[5]. As prior studies showed, angiogenesis was indispensable for providing tumors with nutrients by creating new vascular networks. However, anti-angiogenic therapies targeting VEGF faced challenges due to drug resistance and alternative angiogenic mechanisms, such as vessel co-option.([17,18]) In addition to VEGFA, we further demonstrated that PRKCI also regulates the expression of multiple pro-angiogenic genes, including Ang-2, bFGF, IL-8, MMP9, and VEGFB. Notably, these genes were suppressed in PRKCI knockout cells, upregulated upon PRKCI overexpression, and this induction was abolished by JAK2 S633A mutation. These findings support the broader role of PRKCI in orchestrating a STAT3-driven angiogenic transcriptional program beyond a single effector, reinforcing its central role in tumor angiogenesis regulation. Our study contributed to this field by identifying Prkci as a potential upstream regulator of angiogenesis through its influence on Jak2/Stat3 signaling, thus offering an alternative therapeutic target.

Previous studies highlighted Prkci's role in various cancers, such as osteosarcoma, pancreatic cancer, and cervical cancer. In osteosarcoma, Prkci promoted cell growth via the Akt/mTOR pathway, while in pancreatic cancer, Prkci and RIPK2 jointly enhanced NF-κB/JNK/ERK signaling, driving cancer cell proliferation and invasion[10,13,14]. This study complemented these findings by demonstrating that in CRC, Prkci specifically activated the Jak2/Stat3 pathway, which was known for its involvement in cancer cell survival, proliferation, and angiogenesis. Importantly, our data indicated that Prkci did not alter the overall expression levels of Jak2 or Stat3 but specifically influenced their phosphorylation status, thereby selectively enhancing pathway activation and downstream Vegfa production, which was critical for angiogenic signaling.

One of the most compelling findings of our study was the identification of the Jak2 S633 site as essential for Prkci-mediated Jak2/Stat3 pathway activation. Previous studies had not reported this specific phosphorylation site in the context of Jak2′s role in angiogenesis, making our findings a significant contribution to the understanding of Jak2 regulation in cancer. Mutations at this phosphorylation site (S633A) led to reduced Jak2 activation and downstream Vegfa expression, suggesting a precise regulatory mechanism by which Prkci modulated Jak2/Stat3 signaling and consequently, angiogenesis in CRC. The conservation of this phosphorylation site across species further underscored its functional significance, as demonstrated by our sequence alignment analysis. Although our results strongly support the notion that Prkci regulates Jak2 phosphorylation at S633, a direct biochemical demonstration is currently lacking. In particular, in vitro kinase assays using purified recombinant Prkci and Jak2 proteins were not performed in this study. Therefore, we cannot completely exclude the possibility that Prkci activates Jak2 indirectly through intermediate kinases or scaffold proteins. Nevertheless, our findings—such as the physical interaction between Prkci and Jak2, the loss-of-function and gain-of-function effects of the S633 site, and the ability of phospho-mimetic Jak2 to rescue downstream signaling in Prkci-deficient cells—provide strong mechanistic evidence that Prkci likely acts directly on Jak2 at this residue.

Our findings also aligned with broader literature on Jak2/Stat3 pathway targeting, especially in solid tumors. The Jak2/Stat3 pathway served as a key mediator of oncogenesis and tumor angiogenesis, and its dysregulation was linked to poor prognosis in multiple cancer types[[19], [20], [21]]. This pathway’s role in immune suppression within the tumor microenvironment also supported the potential benefit of targeting Prkci to enhance both anti-angiogenic and immunotherapeutic responses.

Finally, our in vivo xenograft model demonstrated that targeting Prkci significantly reduced tumor growth, angiogenesis, and prolonged survival, supporting the clinical relevance of our findings. These results suggested that Prkci inhibitors could serve as valuable adjuncts to existing anti-angiogenic therapies, potentially overcoming resistance mechanisms associated with VEGF-targeted treatments. Future studies should explore the development of specific inhibitors targeting Prkci or Jak2 phosphorylation at S633 to enhance anti-angiogenesis therapeutic efficacy and mitigate resistance.

Despite the mechanistic insights provided by our study, we acknowledge several limitations. First, although 53 paired colorectal cancer and normal tissues were included, which is consistent with or exceeds sample sizes in comparable translational studies, the sample size remains moderate and warrants cautious interpretation. Second, all clinical tissue samples were collected from medical institutions in Xinjiang, China. While the majority of patients were Han Chinese, a small proportion were from local ethnic minorities such as Uygur and Kazakh. Although no significant differences in Prkci expression were observed between these groups in our internal analysis, the potential for regional or ethnic biological variability cannot be fully excluded. Therefore, the generalizability of our findings may be limited, and future multi-center studies including larger and more diverse populations will be essential to validate our conclusions.

In conclusion, this study identified Prkci as a crucial regulator of angiogenesis in CRC through its activation of the Jak2/Stat3 pathway. Targeting Prkci could offer a novel therapeutic approach for CRC by disrupting tumor angiogenesis and limiting tumor progression.

### Ethics approval and consent to participate

This study involving human participants received approval from the Ethical Committee of People’s Hospital of Xinjiang Uygur Autonomous Region, and written informed consent was obtained from all participants. Animal experiments followed protocols sanctioned by the Institutional Animal Care and Use Committee at People Hospital of Xinjiang Uygur Autonomous Region.

## Consent for publication

All authors have reviewed and agreed to the publication of this manuscript.

## Availability of data and materials

All data supporting the study’s findings are included within the article and its supplementary materials.

## Funding

This research was supported by Xinjiang Uygur Autonomous Regional Science and Technology Support Program(2024E02055); Xinjiang Uygur Autonomous Region Tianshan Talent Training Program (2023TSYCCX0060); 10.13039/100009110Natural Science Foundation of Xinjiang Uygur Autonomous Region (2022D01D77); Xinjiang Uygur Autonomous Region Tianshan Talent Training Program (TSYC202301A031).

## CRediT authorship contribution statement

**Peng Li:** Conceptualization, Data curation, Formal analysis, Writing – original draft. **Guangshi Liu:** Investigation, Formal analysis. **Wenbin Zhang:** Methodology, Data curation, Writing – review & editing. **Tao Li:** Methodology, Data curation, Writing – review & editing. **Xinhui Yang:** Supervision, Visualization, Writing – review & editing.

## Declaration of competing interest

The authors report no competing interests.

## Appendix Supplementary materials

Image, application 1

Image, application 2
