# Supplementary figures and images for: Prkci activates Jak2/Stat3 signaling to promote tumor angiogenesis: Short Name: Prkci in tumor angiogenesis
Source: Neoplasia. 2025 Aug 20;68:101219. doi: 10.1016/j.neo.2025.101219 (PMC12396398; doi:10.1016/j.neo.2025.101219)

Figure S1

A

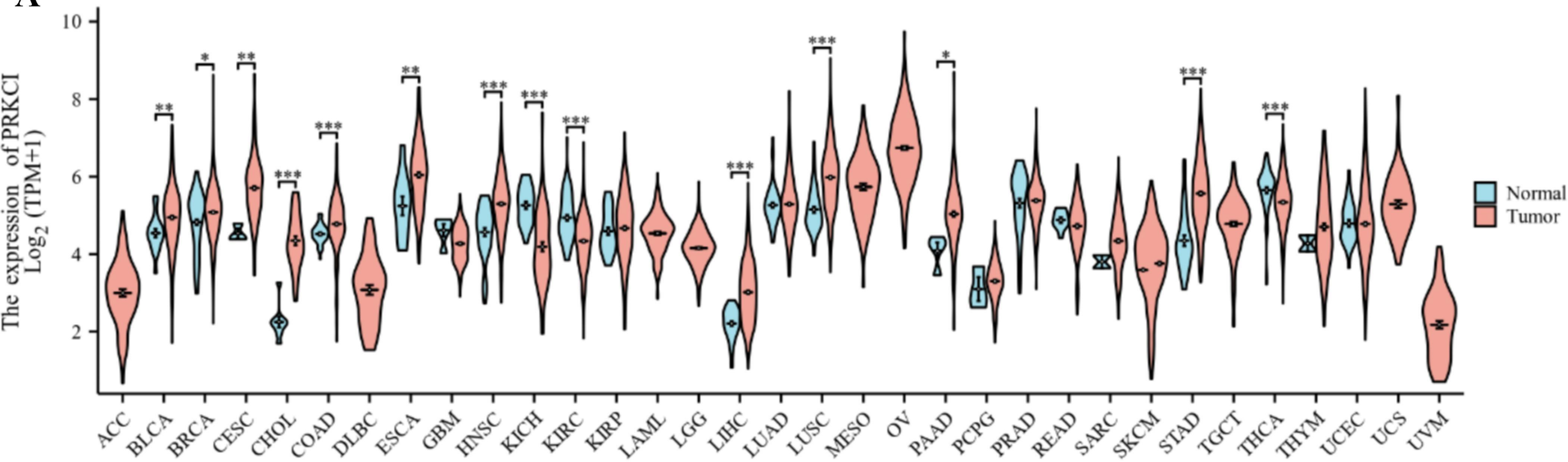

B

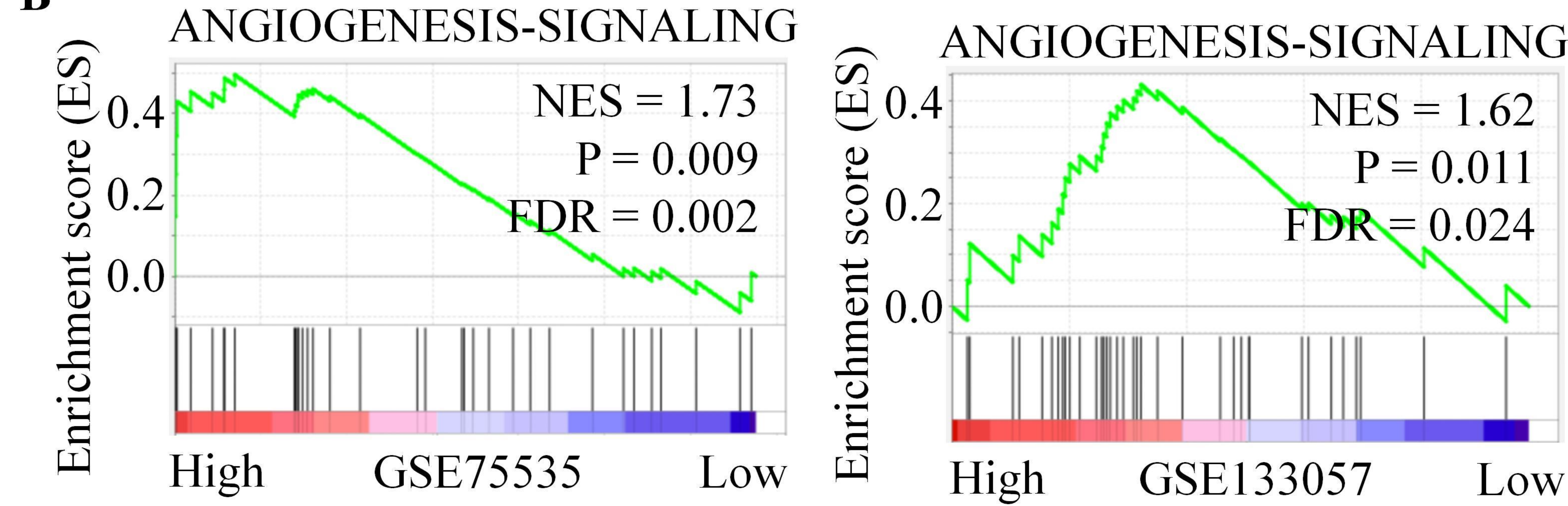

C

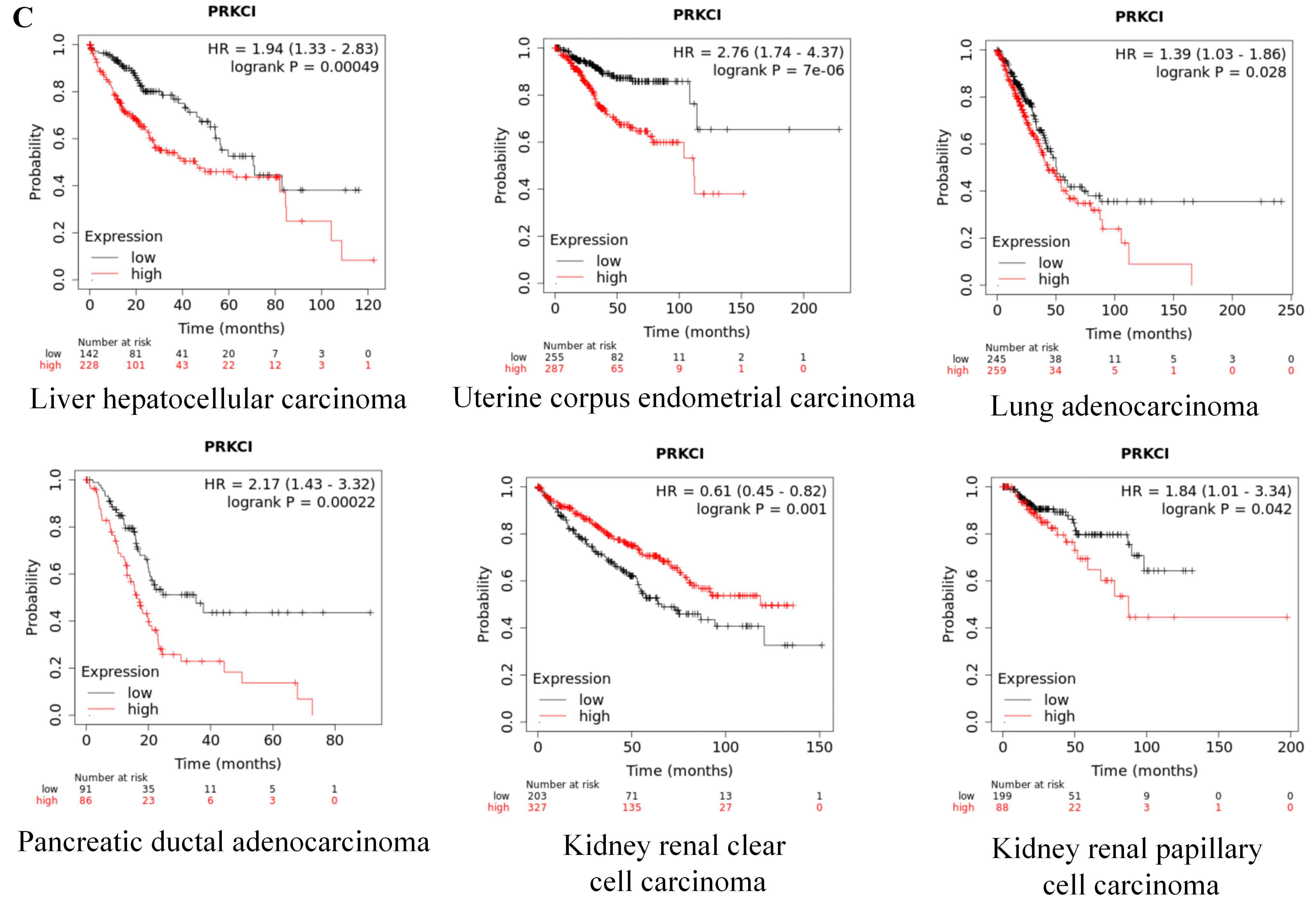

**Figure S2**

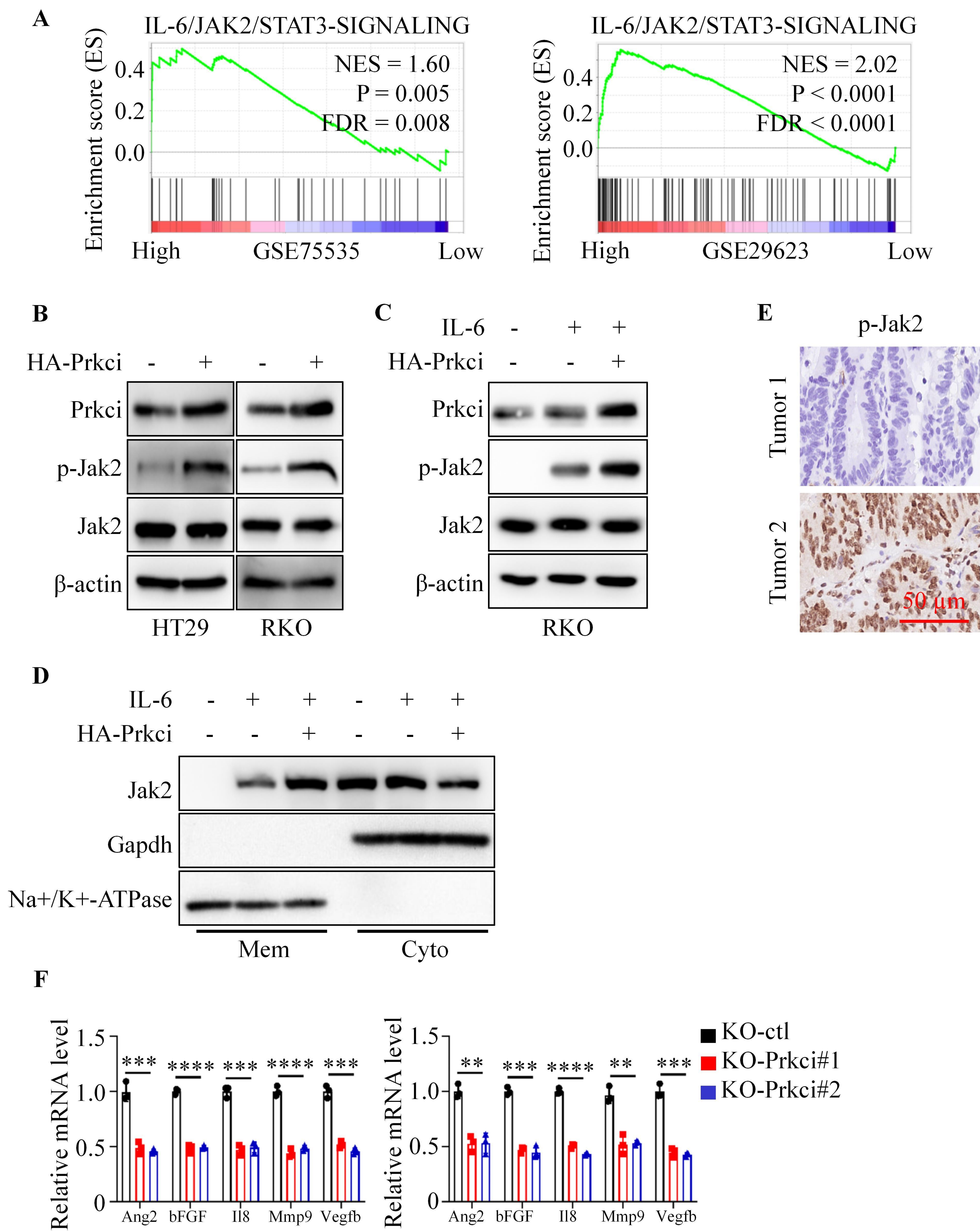

Figure S3

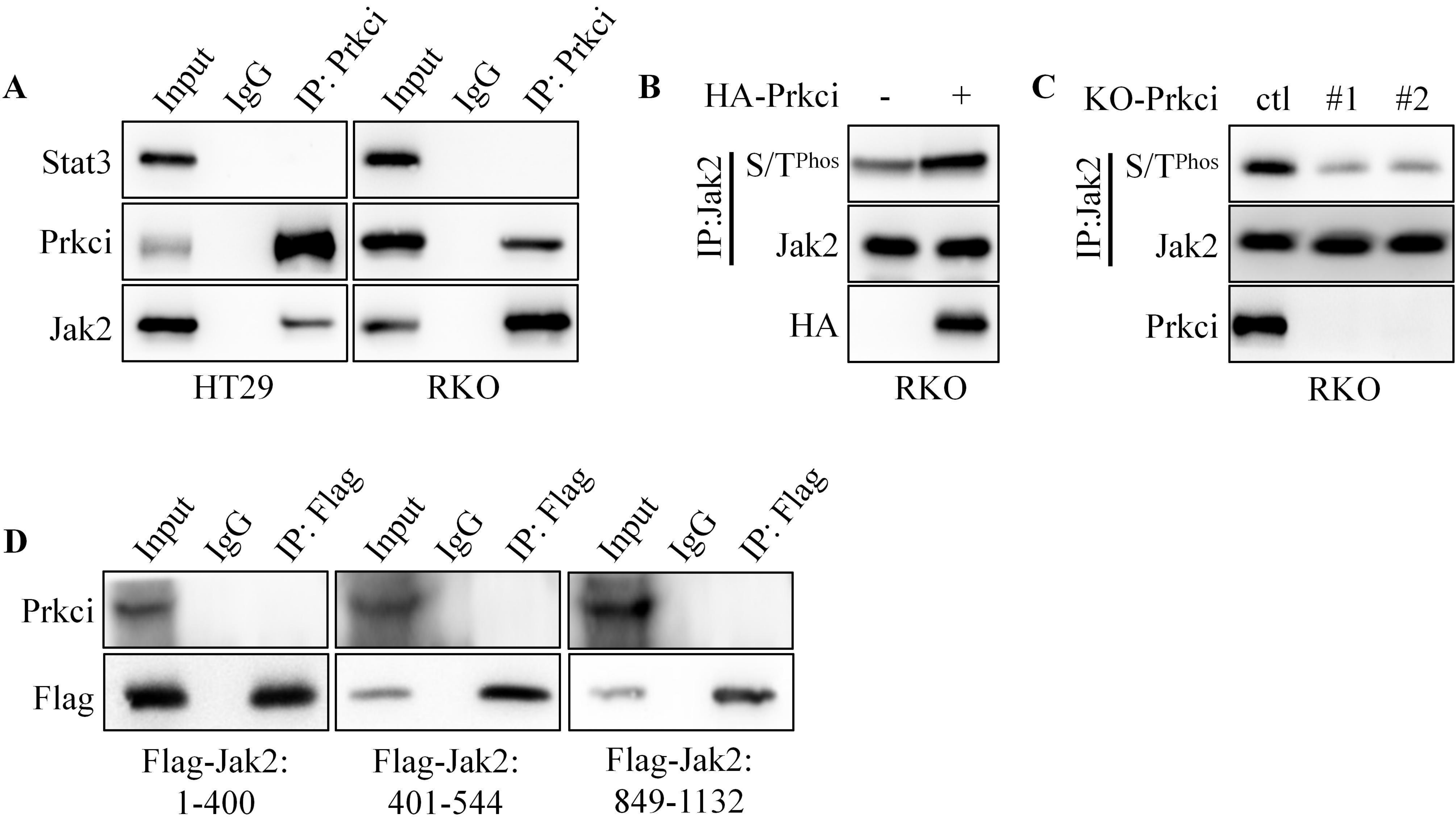

**Figure S4**

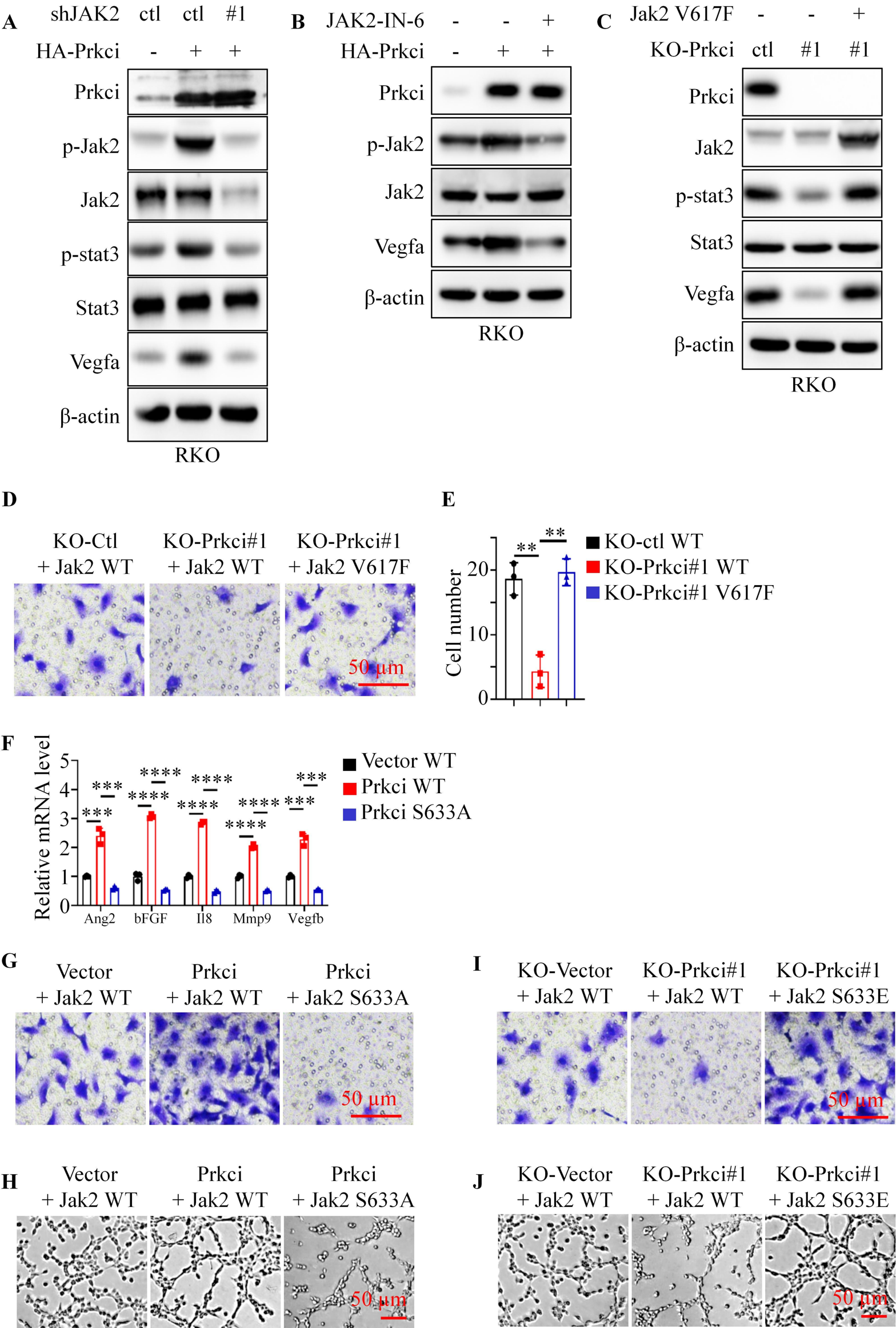

Supplement: Supplementary file 1 [file mmc1.pdf]
