# Supplementary material for: Prkci activates Jak2/Stat3 signaling to promote tumor angiogenesis: Short Name: Prkci in tumor angiogenesis
Source: Neoplasia. 2025 Aug 20;68:101219. doi: 10.1016/j.neo.2025.101219 (PMC12396398; doi:10.1016/j.neo.2025.101219)
Supplement: Supplementary file 2 [file mmc2.docx]

**Supplementary figure legend**

**Supplementary figure legend 1. Prkci expression was elevated in various cancer types and associated with angiogenesis signaling and patient survival.** (A) Violin plots showed significantly higher Prkci expression in multiple tumor types compared to normal tissues. (B) Gene Set Enrichment Analysis (GSEA) demonstrated a positive association between high Prkci expression and angiogenesis signaling. (C) Kaplan-Meier survival curves indicated that high Prkci expression was correlated with poor survival in multiple kinds of cancer.

**Supplementary figure legend 2. Prkci activated the IL-6/Jak2/Stat3 signaling pathway in colorectal cancer cells.** (A) Gene Set Enrichment Analysis (GSEA) showed a positive correlation between high Prkci expression and IL-6/Jak2/Stat3 signaling pathway activation. (B) Western blot analysis indicated that Prkci overexpression increased Jak2 phosphorylation. (C) Prkci overexpression further increased IL-6-induced Jak2 phosphorylation. (D) Membrane and cytoplasmic fractionation analysis demonstrated that Prkci overexpression further upregulated IL-6-induced Jak2 membrane localization. (E) Representative immunohistochemical staining for p-Jak2 in normal and colorectal tumor tissues. (F) Relative mRNA levels of IL6/Jak2/Stat3-targeting genes in KO-ctl and KO-Prkci cells were measured by qPCR. Each IB assay was performed in triplicate, yielding consistent results.

**Supplementary figure legend 3. Prkci interacted with Jak2 and promoted its serine/threonine phosphorylation.** (A) Co-immunoprecipitation (Co-IP) analysis showed that Prkci interacted with Jak2 but not Stat3. (B) Western blot indicated that Prkci overexpression enhanced Jak2 serine/threonine phosphorylation. (C) In KO-Prkci cells, Jak2 S/T^Phos^ was significantly reduced compared to KO-ctl cells. (D) Co-IP analysis detected the binding of Prkci and various Jak2 fragments. Each IB assay was performed in triplicate, yielding consistent results.

**Supplementary figure legend 4. Jak2 was an essential thing for Prkci-mediated tumor angiogenesis.** (A-C) Western blot analysis showed the expression level of multiple proteins in different cells. (D-E) Representative images from trans-well assays, and statistical analysis was shown in Figure S4E. (F) Relative mRNA levels of IL6/Jak2/Stat3-targeting genes in identified cells were measured by qPCR. (G, I) Representative images from trans-well assays. (H, J) Representative images from tube formation assays. Each IB assay was performed in triplicate, yielding consistent results. Statistical analysis was conducted using Student's t-test.
